# Molecular Detection of *Borrelia burgdorferi* s.l. (*Borreliella*) and *Chlamydia*-Like Organism DNA in Early Developmental Stages of Arthropod Vector Species

**DOI:** 10.1155/2023/2511753

**Published:** 2023-10-17

**Authors:** Jiří Petráš, Eva Bártová, Alena Žákovská

**Affiliations:** ^1^Department of Experimental Biology, Faculty of Science, Masaryk University, Kamenice 5, 625 00 Bohunice, Brno, Czech Republic; ^2^Department of Biology and Wildlife Diseases, Faculty of Veterinary Hygiene and Ecology, University of Veterinary Sciences Brno, Palackého tř. 1946/1, 612 42, Brno, Czech Republic; ^3^Department of Biology, Faculty of Education, Masaryk University, Poříčí 7/9, 63900, Brno, Czech Republic

## Abstract

*Borrelia burgdorferi* sensu lato (*Bb*sl) is spirochetes transmitted by ticks and known to cause Lyme disease. *Chlamydia*-like organisms (CLOs) comprise a large group of bacteria that can lead to serious health disorders, including miscarriage. Recently, CLOs have been found in ticks and patient skin biopsies. Due to the involvement of multiple potential vectors in the spread of these pathogens, the objective of this study was to confirm the presence of both organisms in the early developmental stages of selected vectors. Three potential vectors, *Ixodes ricinus* larvae, *Culex pipiens* larvae, and winged (unfed) adults of *Lipoptena cervi*, were collected in the Czech Republic in years 2019–2020. The presence of *Bb*sl and panchlamydial DNA was detected by PCR and positive samples were further analyzed by Sanger sequencing and phylogenetic tree construction. *Bb*sl DNA was proved in 1.5% (2/137) of *I. ricinus* larvae (identified as *Borrelia afzelii* and *Borrelia garinii*), in 1.7% (2/119) of *C. pipiens* larvae (both identified as *B. garinii*), and in 11% (3/27) of *L. cervi* (all identified as *B. garinii*). CLOs were identified in 0.7% (1/137) of *I. ricinus* larvae (Candidatus *Protochlamydia*) and in 7.4% (2/27) of *L. cervi* (unspecified genus), while *C. pipiens* larvae could not be evaluated (0%). This research represents the first investigation of the presence of CLOs in *L. cervi*. The detection of pathogen DNA in the early developmental stages of vectors suggests the potential for transgenerational transmission of *Bb*sl and CLOs in the selected vectors, although at a low rate.

## 1. Introduction

Lyme disease is a serious infection affecting the skeletal, cardiovascular, and nervous systems depending on its form. The late phase of infection may lead to erosive arthritis of larger joints, cardiomyopathy, atrioventricular blockade, meningitis, or cranial neuritis [1, 2]. This disease is caused by Gram-negative bacteria belonging to spirochetes and *Borrelia burgdorferi* sensu lato (*Bb*sl) family, also called *Borreliella* genus [3, 4]. These bacteria are also known for their special abilities, including remarkable movement speed, pleomorphism (for example, turning into a cystic form), or durable biofilm formation [5–7]. All these features allow spirochetes to invade the human immune system or increase bacterial survival in inhospitable conditions. Another attribute of these bacteria is the wide diversity of hosts they can infect, including birds [8, 9], lizards [10], small rodents [11], human patients [12], and ticks [13]. The classical way of transmission of infection through tick bite is well known. The tick feeds on the infected host (usually a small rodent), spirochetes invade the tick gut, then the tick matures and feeds again on an uninfected host and if the period of feeding is long enough, transmission of the pathogen is completed [14]. Transovarial transmission, where the pathogen is passed from an infected female tick to its eggs, was confirmed in the closely related *Borrelia miyamotoi* [15]. It is believed that this mode of infection is also possible in the *Bb*sl group. Since *Bb*sl DNA was detected in the larval stages of our selected vector species [16, 17], the authors considered the potential for transgenerational infection of *Bb*sl, as this way of transmission was previously attributed solely to the *B. miyamotoi* species [18].

*Chlamydia*-like organisms (CLOs) are a group of Gram-negative bacteria, closely related to well-known *Chlamydia* genus. CLOs can inhabit the most diverse types of environments. These bacteria have been found in a large spectrum of hosts, including protozoa [19], insects [20], reptiles [21], fish [22], and humans [23]. Depending on the species, they can cause diseases, including pneumonia, other respiratory disorders, adverse pregnancy outcomes, or even tubal infertility in women [24–28]. These bacteria can be antibiotic resistant on a solitary level [29] and can invade the central part of borrelial biofilm formation. This mutualistic behavior may contribute to their resistance [30]. There is also a similarity between symptoms of some *Borrelia*-caused and *Chlamydia*-caused diseases, for example, both bacterial groups can cause skin manifestations called *erythema nodosum* or *erythema multiforme* [31–33]. Furthermore, *Chlamydia pneumoniae* and IgG antiborrelial antibodies are both often connected to the occurrence of atherosclerotic changes [34, 35] and the DNA of both bacterial groups was detected in synovial fluid of patients with unspecified oligoarthritis [36]. These broadly similar symptoms or suggested mutualistic nature of coinfection may have contributed to the fact that the presence of CLOs in blood-sucking vectors was not known for a long time.

Pathogens like *Bb*sl can be transmitted by multiple vectors. *Ixodes ricinus* is a well-known blood-feeding vector belonging to a vast group of mites. Their three host life cycle (lasting 2–6 years) and wide incidence favor for potential spread of many pathogens, including *Bb*sl. When the larval stage hatches from the egg, it has only six legs and light colored body. After first feeding (typically on a small rodent or bird), it transforms into the nymphal stage with eight legs and dark-colored cuticula. After the second feeding (usually on small or bigger mammals), it follows with final transformation into adults capable of mating. Only female feeds on blood (for nourishment and development of the eggs) and can lay 500–2,000 eggs on the vegetation [37–39]. Mosquitoes of the *Culex* genus belonging to the *Diptera* group are also vectors of large scale of pathogens, including viruses [40], filarial worms [41], and protozoa [42]. Females lay 150–300 eggs in rafts on the surface of stagnant water and these eggs usually hatch within 2 days. Larvae live in water and feed on microscopic organisms and organic matter for about 5 days before developing into the stage of pupa. Pupae stay in water before developing into fully adult individuals within 2–3 days. Both male and female adults feed on nectar or damaged fruit. Fertilized females then need blood as a source of proteins for egg development [43, 44]. *Lipoptena cervi* also belongs to the *Diptera* group and it is a potential vector of pathogens like *Anaplasma* spp. [45], *Bartonella* spp. [46], *Borrelia* spp. [47], or *Trypanosoma* spp. [48]. Although it was stated that there are only specific hosts (wild ruminants) for this vector, the biting of humans was also documented [49]. The life cycle can last 270–370 days, including diapause during winter. The fertilized female produces only one larva, which is inside of her body until pupation. When the pupa is formed, the female drops it from the host to the ground, where it can sink into the snow where it survives through winter. Hatching usually does not start until late summer, then unfed winged hatched adult searches for a suitable host. When an adult attaches to the host, it loses its wings, feeds on the blood, and mates on the host [50–52].

The aim of the study is to determine the presence of *Bb*sl and CLOs in the early developmental stages of selected vectors. The hypothesis is that, given the wide range of host species, the chlamydiaceae, or directly CLO DNA, will be found in *Culex pipiens* and *L. cervi*. This can serve as an initial step toward laying the foundation for future investigations into the potential transovarial transmission of these pathogens, an area that has not received adequate attention in previous research.

## 2. Results

*Bb*sl was identified by PCR in two of 137 (1.5%) *I. ricinus* larvae collected in July 2019. Samples were identified by sequencing analysis as *B. afzelii* and *B. garinii*. *Bb*sl was proved also in two of 119 (1.7%) *C. pipiens* larvae, collected from different reservoirs of stagnant water (both samples characterized as *B. garinii*) and in three of 27 (11.1%) winged adults of *L. cervi* (all characterized as *B. garinii*). An overview of all samples with BLAST similarity is shown in *Supplementary 1*.

**Figure 1:**
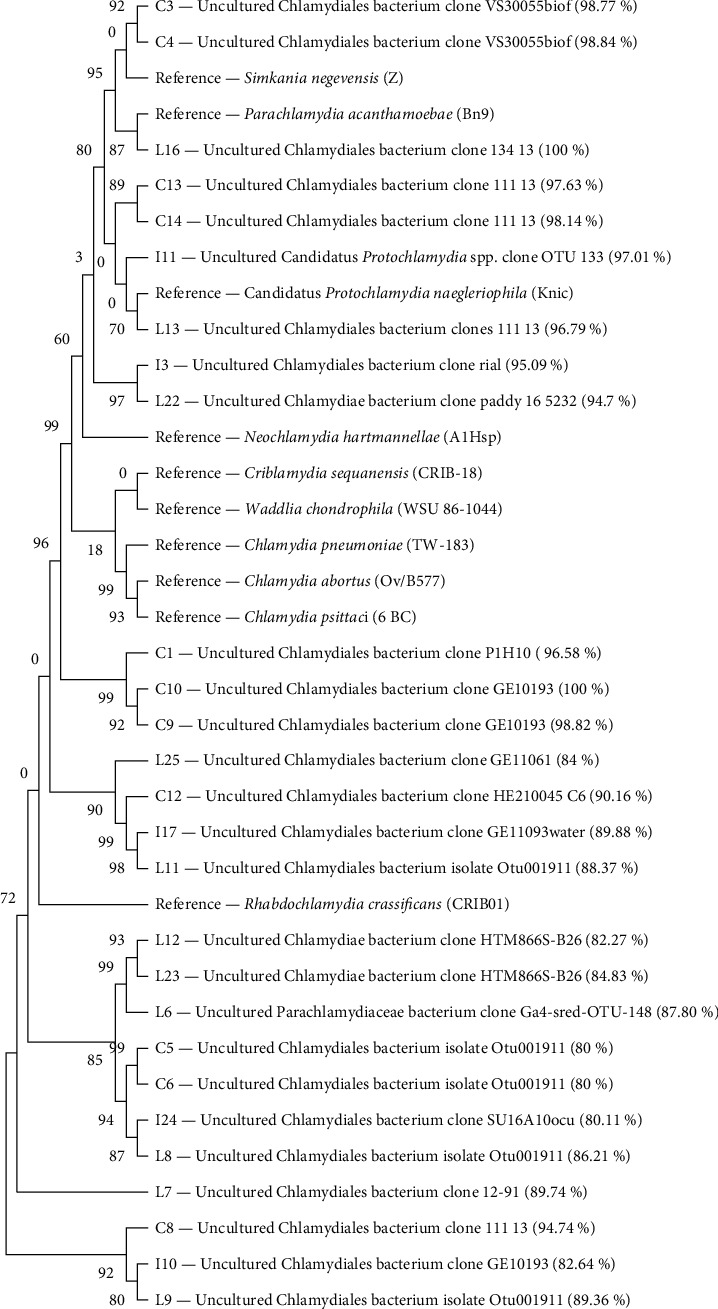
Phylogenetic tree with interior-branch confidence values of Panchlamydia positive samples from all three vectors (BLAST ≥ 80%).

Characterization of CLOs is more complicated; therefore, other two approaches were used. Even with relatively low similarity, Everett et al. [53] used taxonomic cutoffs based on BLAST similarity for *Chlamydia* identification. This approach has been applied to our data (Table 1). According to this approach, only two samples were determined as members of the CLOs group. For further and more precise analysis, the chlamydial phylogenetic tree was constructed with panchlamydial positive samples with BLAST similarity of 80% or higher (Figure 1). For this construction, the referential sequences (three representatives of *Chlamydia* genus, and seven representatives of different CLO genera) found in GenBank were used. This tree correctly connected referential *Protochlamydia* with the analyzed *I. ricinus* sample identified as *Protochlamydia* representative by high phylogenetic proximity. Although a massive prevalence of panchlamydial DNA was detected in *I. ricinus*, only one sample was identified as a CLO representative (Candidatus *Protochlamydia* spp.) with a minimal prevalence of 0.72% (1/137). *C. pipiens* samples (C3 and C4) were most likely closely relative to the *Simkania* genus. Despite many panchlamydial positive *C. pipiens* samples (*Supplementary 1*), no CLO could be identified in this vector species since the samples were collected from the water. Hence, there is no certainty whether the detected panchlamydial DNA originated from *C. pipiens* individuals or from water-residing amoebae. Two *L. cervi* samples (L13 and L16) were phylogenetically paired with *Protochlamydia* and *Parachlamydia*, respectively, therefore belonging to the Parachlamydiaceae family. In contrast, the sample (L6) marked as possible Parachlamydiaceae was cast aside to the lower part of Figure and, therefore, concluded not to be CLO positive. Prevalence of panchlamydial DNA is also relatively high in *L. cervi*; unfortunately, the only sample identified as a Parachlamydiales member by BLAST was not deemed reliable according to the phylogenetic tree (Figure 1). Nevertheless, the phylogenetic tree identified two other samples as representatives of Parachlamydiaceae (2/27; 7.41%).

## 3. Discussion

Every vector sample identified as positive for investigated bacteria was confirmed by PCR at least twice to avoid cross-sample contamination. Further confirmation was carried out by the Sanger sequencing and phylogenetic proximity analysis. Although very low CLO BLAST similarities were found in the pooled samples of *I. ricinus*, particularly around 80%, it is very unlikely that this was due to sample contamination or sequencing errors. The presence of nearly identical single nucleotide polymorphisms (SNPs) within these groups provides evidence that the discrepancies are not the result of sequencing errors or a mixture of random chlamydial DNA in the samples. We conclude that this low similarity is solely attributed to the existence of a vast number of variable chlamydial species that are yet to be discovered, leading to incomplete mapping and registration in GenBank.

With the mentioned incomplete genome mapping and provisional naming of the newly discovered species, it is complicated to determine the taxonomic affiliation of panchlamydial samples. Phylogenetic trees offer great help by connecting samples by evolutionary distance, although these results may rarely contradict the results of Sanger sequencing. We can see this in the example of sample L6, which was identified by Sanger as a close relative to *Parachlamydiaceae* and by Everett's taxonomic standards should belong to Parachlamydiales. However, this sample was put into the lower part of the phylogenetic tree between samples with relatively low BLAST similarity, whereas the *Parachlamydiaceae* representatives were placed in the upper part. We can only assume the reasons behind this, but it is very likely that BLAST similarity below 90% is too low for taxonomic determination. The relatively short length of this sequence (150 bp) may also have directly contributed to the possibility of misevaluation. Another possibility is the potential insufficient purity of the sample after amplicon isolation. On the other hand, sample L6 was placed relatively close to the referential representative of the *Rhabdochlamydia* genus, which is known to be specialized in arthropod infections. This sample was also connected to samples L12 and L23, which do not bear specific order status and are described only as “*Chlamydiae*.” These facts do not exclude the possibility that L6 belongs to the CLO group and may indicate that this sample is closely relative to *Rhabdochlamydiaceae*. Samples C3 and C4 appear to be potentially very close relatives of a referential *Simkania* representative. Unfortunately, samples isolated from *C. pipiens* must be excluded from the CLO determination chapter because the larvae were collected from the water, where CLO-infected protozoa may live and, therefore, devalue these data.

Information about the life cycles of selected three vectors suggests the improbability of acquiring bacteria by feeding on hosts. For example, in *I. ricinus* species, the morphology of larvae indicates that none of these larvae have been fed yet. Several studies also implicate the presence of *Bb*sl and CLOs in *I. ricinus* larvae or even their transovarial transmission, but this transmission is mostly considered inefficient [16, 17, 54–56].

In the case of *C. pipiens*, larvae live in water and do not feed on blood yet in this stage, so there should be no opportunity for them to acquire *Borrelia* by feeding. The presence of borrelial DNA in *C. pipiens* was observed in past studies but with a low incidence [57–59]. Verifying the transmission of CLOs is more complex in this case, as the larvae were collected from water, and it is known that these bacteria can reside within protozoan hosts. Therefore, we exclude the CLO results obtained from *C. pipiens* until it can be confirmed that the identified species of the *Chlamydia* group cannot survive within protozoans. This problem preventing the evaluation of such samples could be solved (at least partially) in several ways. In terms of prevention, it would be advisable, for example, to collect adult mosquitoes and let them lay their eggs in water under laboratory conditions. These eggs would then be stripped of impurities and their DNA isolated. If it is necessary to collect larval individuals, it would be beneficial to evaluate exactly which protozoan species serve CLOs as hosts. After this assessment, samples should then be analyzed by PCR for the presence of DNA of these protozoa. However, this approach does not consider host protozoa that have not yet been shown to have such a relationship with CLO. A similar problem would arise if the DNA of a CLO species that has never been associated with survival in protozoa was found in a sample. There would be no guarantee that such a relationship would not be demonstrated at some point in the future. Taken altogether, the most appropriate approach, in this case, is to collect freshly laid eggs under laboratory conditions.

In *L. cervi*, feeding opportunity is debatable, but since every collected adult was a winged individual, which means it was only in the stage of finding the host, therefore none of them should be fed yet. Also, this species gives us an interesting point of view on positivity, since every female produces only one offspring at a given time, in contrast with the other two investigated vectors. Prevalence of *Bb*sl DNA was reported in representatives of the genus of *Lipoptena* [60], but the transovarial way of transmission was not discussed properly. In contrast, no literature whatsoever on panchlamydial prevalence in *L. cervi* has been found.

Despite the proven presence of the borrelial and chlamydial DNA in vector samples, we cannot deduce if there were viable bacteria. We also cannot possibly know if all these vectors are capable of transmission of these particular bacteria to the hosts; however, this issue could be evaluated by artificially feeding a selected vector on laboratory mice. The viability of these pathogens could be verified by bacterial culture. The crushed and cooled (without freezing) sample would be injected into a culture medium specifically designed for the cultivation of *Bb*sl or *Chlamydiae*. The bacterial species would be further determined by PCR after DNA isolation or by western blotting after protein isolation. However, this approach carries a high risk of media contamination. In the future, the plan is also to test tissue samples from wild rodents for the presence of these pathogens, similar to the work done by part of our collaborative team on *Leptospira* spp. [61].

## 4. Conclusion

We can summarize our data in the following conclusions. DNA of *B. garinii* from the *Bb*sl group was found in all three model organisms, which highly contributes to the theory of its transovarial transmission capability. DNA of CLO representatives was also detected in early developmental stages of *I. ricinus* and *L. cervi*, which contributes to their transgenerational transmission capability. A large number of obstacles have arisen in evaluating (mainly) CLO results of this type of research; a vast amount of chlamydial species, their yet incomplete genome mapping, provisional names, ability to invade protozoans, prevention of sample impurity, and other complications, these are all challenges to be overcome. However, these new results help us to better understand the modes of transmission of some pathogens even in hosts where this was not expected. It also raises new questions about the number of possible hosts/vectors or the number of pathogens themselves in the environment. These unexpectedly high levels of panchlamydial DNA found in the selected model organisms imply that there are likely many more chlamydial species capable of infecting arthropods during their very early developmental stages. Future studies should also consider the viability of these selected pathogens or their ability of transmission between species. If mosquitoes or other species whose life stage involves living in water are to be studied, it is recommended not to isolate DNA from wild larvae but directly from eggs laid in the laboratory environment. Consequently, further investigation into *Borreliella*, Chlamydiales, and Parachlamydiales, including their hosts and modes of transmission, is necessary to gain a deeper understanding of this process.

## 5. Materials and Methods

### 5.1. Sampling

Sampling of *I. ricinus* larvae was done by classical flagging (when a white piece of cloth is drawn through vegetation) in a cottage area and a former wetland called Havřické Vinohrady (49°01′42.0″N 17°36′28.8″E, 250–260 m altitude) near the town of Uherský Brod in Eastern Moravia, Czech Republic in years 2019 (May)–2020 (August). In total, 137 individuals of the larval stage were collected and pooled into 27 samples (divided by the date of collection). Pooled samples contained five individuals on average, with consideration given to isolating individuals from different collections. These representatives were most likely unfed due to their morphology and developmental stage. The samples were frozen and kept at −20°C until DNA isolation.

Sampling of *C. pipiens* larvae was done in a suburban destination near Uherský Brod close to the local river and small brook (49°01′37.9″N 17°37′10.7″E, 201 m altitude). Larval stages were collected from seven distinct reservoirs of stagnant water, stored in tubes, dried, and frozen (−20°C). This set of samples contained a total of 119 individuals pooled in samples of approximately eight individuals. The reservoir number was considered to avoid mixing, and two samples were analyzed from each reservoir.

Sampling of *L. cervi* was done in the same locality by flagging with the modification (white clothes on collector were used to lure the winged adults) to be sure that all collected individuals were winged ones and, therefore, unfed. In total, 27 individuals were collected and separately placed in tubes (one individual per sample) that were frozen and kept at −20°C until DNA isolation.

### 5.2. DNA Isolation and End Point PCR

The DNA was isolated from all frozen samples of vectors using E.Z.N.A.® Insect DNA Kit (Omega Bio-tek, Norcross, USA). Briefly, 2 *µ*l of DNA was mixed with 10 *µ*l EmeraldAmp MAX HS PCR Master Mix (Takara, Shiga, Japan), 7.2 *µ*l PCR water, and 0.8 *µ*l of two primers (Sigma-Aldrich, St. Louis, USA). For *Bb*sl, primers (5′-GTAAGGAAATTAGTTTATGTCTTTT-3′ and 5′-TAAGCTCTTCAAAAAAAGCATCTA-3′) targetting 153 bp fragment of the *hbb* gene were used. It is specific for six typical *Bb*sl genospecies in Europe (*Bb* sensu stricto, *B. afzelii*, *B. garinii*, *Borrelia spielmanii*, *Borrelia lusitaniae*, and *Borrelia valaisiana*). PCR conditions were following: 10 min at 95°C and then 55 cycles (8 s at 95°C, 10 s at 50°C, and 10 s at 72°C). For CLOs, primers (5′-CCGCCAACACTGGGACT-3′ and 5′-GGAGTTAGCCGGTGCTTCTTTAC-3′) targeting 200 bp fragment of 16S rRNA-encoding gene were used. PCR conditions were following: 2 min at 50°C, 10 min at 95°C, and then 45 cycles (15 s at 95°C and 1 min at 60°C) followed by a cooling step at 4°C. PCR products were analyzed by gel electrophoresis. Positive control for *Bb*sl was DNA isolated from *B. burgdorferi* sensu stricto (WSLB 8014/1), *B. garinii* (BRZX 23 MSLB 8064), and *B. afzelii* (BRZX27 MSLB 8065). The DNA isolated from samples C3 and C4 was used as a positive control for CLOs. Every positive sample was confirmed by PCR for the second time and then extracted by PCR DNA Fragments Extraction Kit (Geneaid Biotech, New Taipei City, Taiwan) and sent for sequencing.

### 5.3. Sanger Sequencing and Further Analysis

The concentration and purity of PCR amplicon were measured by NanoDrop ND-1000 (Thermo Fisher Scientific, Waltham, USA) spectrophotometer. The samples and primers were diluted according to Eurofins Genomics (Ebersberg, Germany) requirements and sent for Sanger sequencing. The sequences were checked in SnapGene Viewer and compared with the gene library by nucleotide BLAST search. For the precise analysis of panchlamydial positive samples, taxonomic affiliation was judged according to Everett. This evaluation estimates taxonomic affiliation through developing taxon thresholds based on the GenBank sequence similarity (analyzed by BLAST) [53]. The phylogenetic tree was constructed through MEGA11 software [62]. The evolutionary history was inferred using the neighbor-joining method [63]. The evolutionary distances were computed using the maximum composite likelihood method [64] and are in the units of the number of base substitutions per site. This analysis involved 37 nucleotide sequences. All ambiguous positions were removed for each sequence pair (pairwise deletion option). There were a total of 190 positions in the final dataset.

## Figures and Tables

**Table 1:** Taxonomic identification of chlamydial samples according to Everett's method [53].

Vector	Order (≥80%)	Family (≥90%)	Genus (≥95%)	Species (≥97%)
*I. ricinus*	Chlamydiales (*n* = 3)		ND (*n* = 1)	Candidatus *Protochlamydia* spp. clone OTU_133 (*n* = 1)
*C. pipiens*	Chlamydiales (*n* = 2)	ND (*n* = 2)	ND (*n* = 1)	ND (*n* = 6)
*L. cervi*	Parachlamydiales (*n* = 1)	ND (*n* = 1)	ND (*n* = 1)	ND (*n* = 1)
	Chlamydiales (*n* = 6)			
	ND (*n* = 1)			

*Note*: ND—samples, which cannot be defined on a certain level.

## Data Availability

The data that support the findings of this study are available in the supplementary materials.

## Supplementary Materials

Supplementary 1 Table S1: contains an overview of the prevalence of Borrelial and Panchlamydial DNA in *Ixodes ricinus* samples (BLAST similarity included). Table S2: contains an overview of the prevalence of Borrelial and Panchlamydial DNA in *Culex pipiens* samples (BLAST similarity included). Table S3: contains an overview of the prevalence of Borrelial and Panchlamydial DNA in *Lipoptena cervi* samples (BLAST similarity included). The supplementary data also lists all sequences used, including referential sequences.

Supplementary 2
*Bb*sl sequences—Petras.

Supplementary 3 CLO sequences—Petras.

## References

[1] Girschick H. J., Morbach H., Tappe D. (2009). Treatment of Lyme borreliosis. *Arthritis Research & Therapy*.

[2] Manzoor K., Aftab W., Choksi S., Khan I. A. (2009). Lyme carditis: sequential electrocardiographic changes in response to antibiotic therapy. *International Journal of Cardiology*.

[3] Margos G., Vollmer S. A., Ogden N. H., Fish D. (2011). Population genetics, taxonomy, phylogeny and evolution of *Borrelia burgdorferi* sensu lato. *Infection, Genetics and Evolution*.

[4] Barbour A. G., Adeolu M., Gupta R. S. (2017). Division of the genus *Borrelia* into two genera (corresponding to Lyme disease and relapsing fever groups) reflects their genetic and phenotypic distinctiveness and will lead to a better understanding of these two groups of microbes (Margos, et al. 2016 There is inadequate evidence to support the division of the genus Borrelia. Int. J. Syst. Evol. Microbiol. doi: 10.1099/ijsem.0.001717). *International Journal of Systematic and Evolutionary Microbiology*.

[5] Barbour A. G., Hayes S. F. (1986). Biology of *Borrelia* species. *Microbiological Reviews*.

[6] Sapi E., Bastian S. L., Mpoy C. M. (2012). Characterization of biofilm formation by *Borrelia burgdorferi* in vitro. *PLoS One*.

[7] Meriläinen L., Herranen A., Schwarzbach A., Gilbert L. (2015). Morphological and biochemical features of *Borrelia burgdorferi* pleomorphic forms. *Microbiology*.

[8] McLean R. G., Ubico S. R., Hughes C. A., Engstrom S. M., Johnson R. C. (1993). Isolation and characterization of *Borrelia burgdorferi* from blood of a bird captured in the Saint Croix River Valley. *Journal of Clinical Microbiology*.

[9] Kurtenbach K., Carey D., Hoodless A. N., Nuttall P. A., Randolph S. E. (1998). Competence of pheasants as reservoirs for Lyme disease spirochetes. *Journal of Medical Entomology*.

[10] Swanson K. I., Norris D. E. (2007). Detection of *Borrelia burgdorferi* DNA in lizards from Southern Maryland. *Vector Borne Zoonotic Dis Larchmt N.*.

[11] Levine J. F., Wilson M. L., Spielman A. (1985). Mice as reservoirs of the Lyme disease spirochete. *American Journal of Tropical Medicine and Hygiene*.

[12] Benach J. L., Bosler E. M., Hanrahan J. P. (1983). Spirochetes isolated from the blood of two patients with Lyme disease. *The New England Journal of Medicine*.

[13] Barbour A. G., Burgdorfer W., Hayes S. F., Péter O., Aeschlimann A. (1983). Isolation of a cultivable spirochete fromIxodes ricinus ticks of Switzerland. *Current Microbiology*.

[14] Gern L. (2009). Life cycle of *Borrelia burgdorferi* sensu lato and transmission to humans. *Skin Bioengineering*.

[15] Scoles G. A., Papero M., Beati L., Fish D. (2001). A relapsing fever group spirochete transmitted by *Ixodes scapularis* ticks. *Vector Borne and Zoonotic Diseases (Larchmont, NY)*.

[16] Rijpkema S., Bruinink H. (1996). Detection of *Borrelia burgdorferi* sensu lato by PCR in questing *Ixodes ricinus* larvae from the Dutch North Sea island of Ameland. *Experimental & Applied Acarology*.

[17] van Duijvendijk G., Coipan C., Wagemakers A. (2016). Larvae of *Ixodes ricinus* transmit *Borrelia afzelii* and *B. miyamotoi* to vertebrate hosts. *Parasit Vectors*.

[18] Rollend L., Fish D., Childs J. E. (2013). Transovarial transmission of *Borrelia* spirochetes by *Ixodes scapularis:* a summary of the literature and recent observations. *Ticks Tick-Borne*.

[19] Kahane S., Dvoskin B., Mathias M., Friedman M. G. (2001). Infection of *Acanthamoeba polyphagawith Simkania negevensis* and *S. negevensis* survival within amoebal cysts. *Applied and Environmental Microbiology*.

[20] Thao M. L. L., Baumann L., Hess J. M. (2003). Phylogenetic evidence for two new insect-associated Chlamydia of the family Simkaniaceae. *Current Microbiology*.

[21] Soldati G., Lu Z. H., Vaughan L. (2004). Detection of mycobacteria and chlamydiae in granulomatous inflammation of reptiles: a retrospective study. *Veterinary Pathology*.

[22] Fehr A., Walther E., Schmidt-Posthaus H. (2013). *Candidatus Syngnamydia venezia*, a novel member of the phylum Chlamydiae from the broad nosed pipefish, *Syngnathus typhle*. *PloS One*.

[23] Kahane S., Greenberg D., Friedman M. G., Haikin H., Dagan R. (1998). High prevalence of Simkania Z, a novel Chlamydia-like bacterium, in infants with acute bronchiolitis. *Journal of Infectious Diseases*.

[24] Greub G., Boyadjiev I., La Scola B., Raoult D., Martin C. (2003). Serological hint suggesting that Parachlamydiaceae are agents of pneumonia in polytraumatized intensive care patients. *Annals of the New York Academy of Sciences*.

[25] Baud D., Thomas V., Arafa A., Regan L., Greub G. (2007). Waddlia chondrophila, a potential agent of human fetal death. *Emerging Infectious Diseases*.

[26] Borel N., Ruhl S., Casson N., Kaiser C., Pospischil A., Greub G. (2007). *Parachlamydia* spp. and related Chlamydia-like organisms and bovine abortion. *Emerging Infectious Diseases*.

[27] Lamoth F., Jaton K., Vaudaux B., Greub G. (2011). Parachlamydia and Rhabdochlamydia: emerging agents of community-acquired respiratory infections in children. *Clinical Infectious Diseases: An Official Publication of the Infectious Diseases Society of America*.

[28] Verweij S. P., Kebbi-Beghdadi C., Land J. A., Ouburg S., Morré S. A., Greub G. (2015). *Waddlia chondrophila* and *Chlamydia trachomatis* antibodies in screening infertile women for tubal pathology. *Microbes and Infection*.

[29] Somani J., Bhullar V. B., Workowski K. A., Farshy C. E., Black C. M. (2000). Multiple drug-resistant *Chlamydia trachomatis* associated with clinical treatment failure. *Journal of Infectious Diseases*.

[30] Sapi E., Gupta K., Wawrzeniak K. (2019). *Borrelia* and *Chlamydia* can form mixed biofilms in infected human skin tissues. *European Journal of Microbiology and Immunology*.

[31] Kousa M., Saikku P., Kanerva L. (1980). Erythema nodosum in chlamydial infections. *Acta Dermato-Venereologica*.

[32] Schuttelaar M. L., Laeijendecker R., Heinhuis R. J., Van Joost T. (1997). Erythema multiforme and persistent erythema as early cutaneous manifestations of Lyme disease. *Journal of the American Academy of Dermatology*.

[33] Simakova A. I., Popov A. F., Dadalova O. B. (2005). Ixodes tick-borne borreliosis with erythema nodosum. *Meditsinskaia parazitologiia i parazitarnye bolezni*.

[34] Valtonen V. V. (1991). Infection as a risk factor for infarction and atherosclerosis. *Annals of Medicine*.

[35] Völzke H., Wolff B., Lüdemann J. (2006). Seropositivity for anti-Borrelia IgG antibody is independently associated with carotid atherosclerosis. *Atherosclerosis*.

[36] Schnarr S., Putschky N., Jendro M. C. (2001). *Chlamydia* and *Borrelia* DNA in synovial fluid of patients with early undifferentiated oligoarthritis: results of a prospective study. *Arthritis and Rheumatism*.

[37] Capinera J. L. (2008). *Encyclopedia of Entomology*.

[38] Stanek G., Wormser G. P., Gray J., Strle F. (2012). Lyme borreliosis. *Lancet*.

[39] Sonenshine D. E., Roe R. M. (2013). *Biology of Ticks*.

[40] Reisen W. K., Milby M. M., Presser S. B., Hardy J. L. (1992). Ecology of mosquitoes and St. Louis encephalitis virus in the Los Angeles Basin of California, 1987–1990. *Journal of Medical Entomology*.

[41] Sabatinelli G., Ranieri E., Gianzi F. P., Papakay M., Cancrini G. (1994). Role of *Culex quinquefasciatus* in the transmission of bancroftian filariasis in the Federal Islamic Republic of Comoros (Indian Ocean). *Parasite Paris France*.

[42] Kimura M., Darbro J. M., Harrington L. C. (2010). Avian malaria parasites share congeneric mosquito vectors. *Journal of Parasitology*.

[43] Vinogradova E. B. (2000). *Culex Pipiens* Pipiens Mosquitoes: Taxonomy, Distribution, Ecology, Physiology, Genetics, Applied Importance and Control.

[44] Foster W., Walker E. (2002). Mosquitoes (Culicidae). *Medical and Veterinary Entomology*.

[45] Hornok S., de la Fuente J., Biró N. (2011). First molecular evidence of Anaplasma ovis and Rickettsia spp. in keds (Diptera: Hippoboscidae) of sheep and wild ruminants. *Vector Borne and Zoonotic Diseases (Larchmont, NY)*.

[46] de Bruin A., van Leeuwen A. D., Jahfari S. (2015). Vertical transmission of *Bartonella schoenbuchensis* in *Lipoptena cervi*. *Parasit Vectors*.

[47] Buss M., Case L., Kearney B., Coleman C., Henning J. D. (2016). Detection of Lyme disease and anaplasmosis pathogens via PCR in Pennsylvania deer ked. *Journal of Vector Ecology*.

[48] Böse R., Petersen K. (2004). *Lipoptena cervi* (Diptera), a potential vector of *Megatrypanum* trypanosomes of deer (Cervidae). *Parasitology Research*.

[49] Bezerra-Santos M. A., Otranto D. (2020). Keds, the enigmatic flies and their role as vectors of pathogens. *Acta Tropica*.

[50] Kaitala A., Kortet R., Härkönen S. (2009). Deer ked, an ectoparasite of moose in Finland: a brief review of its biology and invasion. *Alces*.

[51] Paakkonen T., Mustonen A. M., Roininen H., Niemelä P., Ruusila V., Nieminen P. (2010). Parasitism of the deer ked, Lipoptena cervi, on the moose, Alces alces, in eastern Finland. *Medical and Veterinary Entomology*.

[52] Korhonen E. M., Vera C. Pérez, Pulliainen A. T. (2015). Molecular detection of *Bartonella* spp. in deer ked pupae, adult keds and moose blood in Finland. *Epidemiology and Infection*.

[53] Everett K. D., Bush R. M., Andersen A. A. (1999). Emended description of the order Chlamydiales, proposal of Parachlamydiaceae fam. nov. and Simkaniaceae fam. nov., each containing one monotypic genus, revised taxonomy of the family Chlamydiaceae, including a new genus and five new species, and standards for the identification of organisms. *International Journal of Systematic Bacteriology*.

[54] Rijpkema S., Nieuwenhuijs J., Franssen F. F., Jongejan F. (1994). Infection rates of *Borrelia burgdorferi* in different instars of *Ixodes ricinus* ticks from the Dutch North Sea Island of Ameland. *Experimental & Applied Acarology*.

[55] Hokynar K., Sormunen J. J., Vesterinen E. J. (2016). Chlamydia-like organisms (CLOs) in finnish *Ixodes ricinus* ticks and human skin. *Microorganisms*.

[56] Hauck D., Jordan D., Springer A. (2020). Transovarial transmission of *Borrelia* spp., *Rickettsia* spp. and *Anaplasma phagocytophilum* in *Ixodes ricinus* under field conditions extrapolated from DNA detection in questing larvae. *Parasit Vectors*.

[57] Žákovská A., Nejedla P., Holíková A., Dendis M. (2002). Positive findings of *Borrelia burgdorferi* in *Culex* (Culex) pipiens pipiens larvae in the surrounding of Brno city determined by the PCR method. *Annals of Agricultural and Environmental Medicine (AAEM)*.

[58] Nejedla P., Norek A., Vostal K., Zakovska A. (2009). What is the percentage of pathogenic borreliae in spirochaetal findings of mosquito larvae?. *Annals of Agricultural and Environmental Medicine (AAEM)*.

[59] Melaun C., Zotzmann S., Santaella V. G. (2016). Occurrence of *Borrelia burgdorferi* s.l in different genera of mosquitoes (Culicidae) in Central Europe. *Ticks Tick-Borne Diseases*.

[60] Gałęcki R., Jaroszewski J., Bakuła T., Galon E. M., Xuan X. (2021). Molecular detection of selected pathogens with zoonotic potential in deer keds (*Lipoptena fortisetosa*). *Pathogens (Basel, Switzerland)*.

[61] Žákovská A., Treml F., Nejezchlebová H., Nepeřený J., Budíková M., Bártová E. (2022). *Leptospira interrogans* Sensu Lato in wild small mammals in three moravian localities of the Czech Republic. *Pathogens*.

[62] Tamura K., Stecher G., Kumar S. (2021). Molecular evolutionary genetics analysis version 11. *Molecular Biology and Evolution*.

[63] Saitou N., Nei M. (1987). The neighbor-joining method: a new method for reconstructing phylogenetic trees. *Molecular Biology and Evolution*.

[64] Tamura K., Nei M., Kumar S. (2004). Prospects for inferring very large phylogenies by using the neighbor-joining method. *Proceedings of the National Academy of Sciences*.

