# Supplementary material for: Molecular Detection of Borrelia burgdorferi s.l. (Borreliella) and Chlamydia-Like Organism DNA in Early Developmental Stages of Arthropod Vector Species
Source: Transbound Emerg Dis. 2023 Oct 17;2023:2511753. doi: 10.1155/2023/2511753 (PMC12017234; doi:10.1155/2023/2511753)
Supplement: Supplementary 1 — Table S1: contains an overview of the prevalence of Borrelial and Panchlamydial DNA in Ixodes ricinus samples (BLAST similarity included). Table S2: contains an overview of the prevalence of Borrelial and Panchlamydial DNA in Culex pipiens samples (BLAST similarity included). Table S3: contains an overview of the prevalence of Borrelial and Panchlamydial DNA in Lipoptena cervi samples (BLAST similarity included). The supplementary data also lists all sequences used, including referential sequences. [file 2511753.f1.docx]

**Table S1.** An overview of the prevalence of Borrelial and Panchlamydial DNA in 137 *Ixodes ricinus* larvae (BLAST similarity included)

| ***Ixodes ricinus*** | | | | |
| --- | --- | --- | --- | --- |
| Sample | No. of individuals | Date | *Bb*sl | Panchlamydia |
| I1 | 2 | 08.05.2019 |  | Uncultured Chlamydiales bacterium clone 13-03 (77.69%) |
| I2 | 6 | 13.05.2019 |  | Uncultured Chlamydiales bacterium isolate Otu001911 (78.71%) |
| I3 | 2 | 21.05.2019 |  | ***Uncultured Chlamydiales bacterium clone rial (95.09%)** |
| I4 | 6 | 31.05.2019 |  | Uncultured Chlamydiales bacterium isolate Otu001911 (78.71%) |
| I5 | 7 | 10.06.2019 |  | Uncultured Chlamydiales bacterium isolate Otu001911 (79.22%) |
| I6 | 1 | 10.06.2019 |  | Uncultured Chlamydiales bacterium isolate Otu001911 (78.71%) |
| I7 | 5 | 26.06.2019 |  | Failed |
| I8 | 6 | 26.06.2019 |  | Uncultured Chlamydiales bacterium isolate Otu001911 (79.49%) |
| I9 | 6 | 08.07.2019 |  | Uncultured Chlamydiales bacterium isolate Otu001911 (78.71%) |
| I10 | 6 | 08.07.2019 |  | Uncultured Chlamydiales bacterium clone GE10193 (82.64%) |
| I11 | 6 | 08.07.2019 | ***B. afzelii* (100%)** | ****Uncultured Candidatus *Protochlamydia* sp. clone OTU_133 (97.01%)** |
| I12 | 7 | 08.07.2019 |  | Uncultured Chlamydiales bacterium clone SU16A10ocu (78.74%) |
| I13 | 6 | 16.07.2019 |  | Uncultured Chlamydiales bacterium clone SU16A10ocu (79.19%) |
| I14 | 6 | 16.07.2019 |  | Uncultured Chlamydiales bacterium isolate Otu001911 (78.71%) |
| I15 | 6 | 16.07.2019 |  | Uncultured Chlamydiales bacterium isolate Otu001911 (78.71%) |
| I16 | 4 | 16.07.2019 |  | Uncultured Chlamydiales bacterium clone SU16A10ocu (79.53%) |
| I17 | 4 | 23.07.2019 | ***B. garinii* (100%)** | Uncultured Chlamydiales bacterium clone GE11093water (89.88%) |
| I18 | 4 | 23.07.2019 |  | Uncultured Chlamydiales bacterium isolate Otu001911 (79.35%) |
| I19 | 6 | 29.07.2019 |  | Uncultured Chlamydiales bacterium clone 12-108 (77.94%) |
| I20 | 6 | 29.07.2019 |  | Uncultured Chlamydiales bacterium isolate Otu001911 (77.40%) |
| I21 | 5 | 29.07.2019 |  | Uncultured Chlamydiales bacterium isolate Otu001911 (79.49%) |
| I22 | 6 | 19.08.2019 |  | Uncultured Chlamydiales bacterium isolate Otu001911 (78.98%) |
| I23 | 5 | 19.08.2019 |  | Uncultured Chlamydiales bacterium isolate Otu001911 (78.98%) |
| I24 | 3 | 26.08.2019 |  | Uncultured Chlamydiales bacterium clone SU16A10ocu (80.11%) |
| I25 | 1 | 26.10.2019 |  | Uncultured Chlamydiales bacterium clone HE20036biof (77.38%) |
| I26 | 7 | 21.08.2020 |  | Uncultured Chlamydiales bacterium clone SU16A10ocu (79.65%) |
| I27 | 6 | 23.08.2020 |  | Uncultured Chlamydiales bacterium clone SU16A10ocu (79.65%) |

***Bold font marks samples with BLAST similarity ~95 % and above.**

****Green highlighting marks samples recognized by BLAST as CLO positive.**

**Table S2.** An overview of the prevalence of Borrelial and Panchlamydial DNA in 119 *Culex pipiens* larvae (BLAST similarity included)

| ***Culex pipiens pipiens*** | | | | | |
| --- | --- | --- | --- | --- | --- |
| Sample | Reservoir | No. of individuals | Date | *Bb*sl | Panchlamydia |
| C1 | 1 | 10 | 31.08.2020 |  | **Uncultured *Chlamydiales* bacterium clone P1H10 ( 96.58%)** |
| C2 | 1 | 10 | 31.08.2020 | ***B. garinii* (99.13%)** |  |
| C3 | 2 | 6 | 21.10.2020 | ***B. garinii* (99.09%)** | **Uncultured Chlamydiales bacterium clone VS30055biof (98.77%)** |
| C4 | 2 | 4 | 21.10.2020 |  | **Uncultured Chlamydiales bacterium clone VS30055biof (98.84%)** |
| C5 | 3 | 10 | 21.10.2020 |  | Uncultured Chlamydiales bacterium isolate Otu001911 (80%) |
| C6 | 3 | 10 | 21.10.2020 |  | Uncultured Chlamydiales bacterium isolate Otu001911 (80%) |
| C7 | 4 | 7 | 21.10.2020 |  |  |
| C8 | 4 | 6 | 21.10.2020 |  | **Uncultured Chlamydiales bacterium clone 111_13 (94.74%)** |
| C9 | 5 | 7 | 21.10.2020 |  | **Uncultured Chlamydiales bacterium clone GE10193 (98.82%)** |
| C10 | 5 | 7 | 21.10.2020 |  | **Uncultured Chlamydiales bacterium clone GE10193 (100%)** |
| C11 | 6 | 9 | 21.10.2020 |  |  |
| C12 | 6 | 9 | 21.10.2020 |  | Uncultured Chlamydiales bacterium clone HE210045_C6 (90.16%) |
| C13 | 7 | 12 | 27.10.2020 |  | **Uncultured Chlamydiales bacterium clone 111_13 (97.63%)** |
| C14 | 7 | 12 | 27.10.2020 |  | **Uncultured Chlamydiales bacterium clone 111_13 (98.14%)** |

**Table S3.** An overview of the prevalence of Borrelial and Panchlamydial DNA in 27 *Lipoptena cervi* winged adults (BLAST similarity included)

| ***Lipoptena cervi*** | | | | |
| --- | --- | --- | --- | --- |
| Sample | No. of individuals | Date | *Bb*sl | Panchlamydia |
| L1 | 1 | 30.08.2020 |  |  |
| L2 | 1 | 30.08.2020 |  |  |
| L3 | 1 | 30.08.2020 |  |  |
| L4 | 1 | 30.08.2020 | ***B. garinii* (97.48%)** |  |
| L5 | 1 | 30.08.2020 |  | Uncultured Chlamydiales bacterium isolate Otu001911 (78.71%) |
| L6 | 1 | 30.08.2020 |  | Uncultured *Parachlamydiaceae* bacterium clone Ga4-sred-OTU-148 (87.80%) |
| L7 | 1 | 30.08.2020 |  | Uncultured Chlamydiales bacterium clone 12-91 (89.74%) |
| L8 | 1 | 30.08.2020 |  | Uncultured Chlamydiales bacterium isolate Otu001911 (86.21%) |
| L9 | 1 | 30.08.2020 |  | Uncultured Chlamydiales bacterium isolate Otu001911 (89.36%) |
| L10 | 1 | 30.08.2020 |  |  |
| L11 | 1 | 30.08.2020 |  | Uncultured Chlamydiales bacterium isolate Otu001911 (88.37%) |
| L12 | 1 | 30.08.2020 |  | Uncultured Chlamydiae bacterium clone HTM866S-B26 (82.27%) |
| L13 | 1 | 07.09.2020 | ***B. garinii* (96.58%)** | **Uncultured Chlamydiales bacterium clone 111_13 (96.79%)** |
| L14 | 1 | 07.09.2020 |  |  |
| L15 | 1 | 07.09.2020 |  |  |
| L16 | 1 | 07.09.2020 |  | **Uncultured Chlamydiales bacterium clone 134_13 (100%)** |
| L17 | 1 | 07.09.2020 |  |  |
| L18 | 1 | 07.09.2020 |  |  |
| L19 | 1 | 07.09.2020 | ***B. garinii* (99.15%)** |  |
| L20 | 1 | 07.09.2020 |  |  |
| L21 | 1 | 07.09.2020 |  | Uncultured Chlamydiae bacterium clone HTM866S-B26 (77.27%) |
| L22 | 1 | 07.09.2020 |  | **Uncultured Chlamydiae bacterium clone Paddy_16_5232 (94.7%)** |
| L23 | 1 | 07.09.2020 |  | Uncultured Chlamydiae bacterium clone HTM866S-B26 (84.83%) |
| L24 | 1 | 07.09.2020 |  |  |
| L25 | 1 | 07.09.2020 |  | Uncultured Chlamydiales bacterium clone GE11061 (84%) |
| L26 | 1 | 07.09.2020 |  |  |
| L27 | 1 | 07.09.2020 |  |  |
