# Supplementary material for: Molecular Detection of Borrelia burgdorferi s.l. (Borreliella) and Chlamydia-Like Organism DNA in Early Developmental Stages of Arthropod Vector Species
Source: Transbound Emerg Dis. 2023 Oct 17;2023:2511753. doi: 10.1155/2023/2511753 (PMC12017234; doi:10.1155/2023/2511753)
Supplement: Supplementary 2 — Bbsl sequences—Petras. [file 2511753.f2.docx]

>Reference - Borrelia burgdorferi sensu stricto (P1G)

ATGTCTTTTTCAAGAAGACCAAAGGTTACTAAGTCAGACATTGTTGATCAAATATCTTTGAATATTAAAAATAATAATCTGAAATTAGAAAAAAAATACATAAGACTTGTAATAGATGCTTTTTTTGAAGAGCTTAA

>Reference - Borrelia burgdorferi sensu stricto (IP3)

ATGTCTTTTTCAAGAAGACCAAAGGTTACTAAGTCAGACATTGTTGATCAAATATCTTTGAATATTAAAAATAATAATCTGAAATTAGAAAAAAAATACATAAGACTTGTAATAGATGCTTTTTTTGAAGAGCTTAA

>Reference - Borrelia garinii (PBi)

ATGTCTTTTTCAAGAAGACCGAAGATTACTAAATCAGATATTGTTGATCAAATATCTTTGAATATTAGAAATAATAATCTAAAATTAGAAAAAAAATACATAAGGCTCGTAATAGATGCTTTTTTTGAAGAGCTTAA

>Reference - Borrelia garinii (VS102)

ATGTCTTTTTCAAGAAGACCGAAGATTACTAAATCAGATATTGTTGATCAAATATCTTTGAATATTAGAAATAATAATCTAAAATTAGAAAAAAAATACATAAGACTCGTAATAGATGCTTTTTTTGAAGAGCTTAA

>Reference - Borrelia afzelii (BO23)

ATGTCTTTTCCAAGAAGACCAAAGGTTACTAAGTCAGATATTGTTGATCAAATATCTTTGAATATTAGAAATAATAATCTAAAACTAGAAAAAAAATACATAAGACTTGTAATAGATGCTTTTTTTGAAGAGCTTAA

>Reference - Borrelia afzelii (VS461)

ATGTCTTTTCCAAGAAGACCAAAGGTTACTAAGTCAGATATTGTTGATCAAATATCTTTGAATATTAGAAATAATAATCTAAAACTAGAAAAAAAATATATAAGACTTGTAATAGATGCTTTTTTTGAAGAGCTTAA

>Reference - Borrelia spielmanii (A14S)

ATGTCTTTTTCAAGAAGACCAAAGATTACTAAGTCAGATATTGTTGATCAAATATCTTTAAATATTAGAAATAATAATCTAAAACTAGAAAAAAAATACATAAGACTTGTAATAGATGCTTTTTTTCAAGAGCTTAA

>Reference - Borrelia spielmanii (PZ30802)

ATGTCTTTTTCAAGAAGACCAAAGATTACTAAGTCAGATATTGTTGATCAAATATCTTTAAATATTAGAAATAATAATCTAAAACTAGAAAAAAAATACATAAGACTTGTAATAGATGCTTTTTTTCAAGAGCTTAA

>Reference - Borrelia valaisiana (Am501)

ATGTCTTTTCCAAGAAGACCAAAGATTACTAAGTCGGATATTGTTGATCAAATATCTTTGAATATTAGAAATAATAATCTAAAATTAGAAAAAAAATACATAAGACTTGTAATAGATGCTTTTTTTGAAGAGCTTAA

>Reference - Borrelia valaisiana (M7)

ATGTCTTTTCCAAGAAGACCAAAGATTACTAAGTCAGATATTGTTGATCAAATATCTTTGAATATTAGAAATAATAATCTAAAATTAGAAAAAAAATACATAAGACTTGTAATAGATGCTTTTTTTGAAGAGCTTAA

>Reference - Borrelia lusitaniae (RB-La1N1)

ATGTCTTTTCCAAGAAGGCCAAAGGTTACTAAGTCAGATGTTGTTAATCAAATATCTTTAAATATTAGAAATAATAATCTAAAGCTAGAAAAAAAATACATAAGACTTGTAATAGATGCTTTTTTTGAAGAGCTTAA

>Reference - Borrelia lusitaniae (RB-Pm2N6)

ATGTCTTTTCCAAGAAGGCCAAAGGTTACTAAGTCAGATGTTGTTAATCAAATATCTTTAAATATTAGAAATAATAATCTAAAGCTAGAAAAAAAATACATAAGACTTGTAATAGATGCTTTTTTTGAAGAGCTTAA

>C2 - Borrelia garinii (99.13 percent)

---------------AATGACGAGGATACTAAATCAGATATTGTTGATCAAATATCTTTGAATATTAGAAATAATAATCTAAAATTAGAAAAAAAATACATAAGACTCGTAATAGATGCTTTTTTTGAAGAGCTTAA

>C3 - Borrelia garinii (99.09 percent)

-----------------CTAGCGGAGATACTAATCAGATATTGTTGATCAAATATCTTTGAATATTAGAAATAATAATCTAAAATTAGAAAAAAAATACATAAGACTCGTAATAGATGCTTTTTTTGAAGAGCTTAG

>I11 - Borrelia afzelii (100 percent)

----------CAAAGAACCCAAAGGTTACTAAGTCAGATATTGTTGATCAAATATCTTTGAATATTAGAAATAATAATCTAAAACTAGAAAAAAAATACATAAGACTTGTAATAGATGCTTTTTTTGAAGAGCTTAA

>I17 - Borrelia garinii (100 percent)

--------------ACTAGCCGAGATTACTAAATCAGATATTGTTGATCAAATATCTTTGAATATTAGAAATAATAATCTAAAATTAGAAAAAAAATACATAAGACTCGTAATAGATGCTTTTTTTGAAGAGCTTAG

>L4 - Borrelia garinii (97.48 percent)

---------GAACTGAGACGGAGGATTACTAAATCAGATATTGTTGATCAAATATCTTTGAATATTAGAAATAATAATCTAAAATTAGAAAAAAAATACATAAGACTCGTAATAGATGCTTTTTTTGAAGGGCTAGG

>L13 - Borrelia garinii (96.58 percent)

---------AAATAAAACGGGAAGATTACTAAATCAGATATTGTTGAGCAAATATCCTTGAGGATTAGAAATAATAATCTAAAATTAGAAAAAAAATACATAAGACTCGTAATAGATGCTTTTTTTGAAGAGCTTAA

>L19 - Borrelia garinii (99.15 percent)

-------------------GGAGGATTACTAAATCAGATATTGTTGATCAAATATCTTTGAATATTAGAAATAATAATCTAAAATTAGAAAAAAAATACATAAGACTCGTAATAGATGCTTTTTTTGAAGAGCTTAA
