# Supplementary material for: Molecular Detection of Borrelia burgdorferi s.l. (Borreliella) and Chlamydia-Like Organism DNA in Early Developmental Stages of Arthropod Vector Species
Source: Transbound Emerg Dis. 2023 Oct 17;2023:2511753. doi: 10.1155/2023/2511753 (PMC12017234; doi:10.1155/2023/2511753)
Supplement: Supplementary 3 — CLO sequences—Petras. [file 2511753.f3.docx]

>Reference - Chlamydia pneumoniae (TW-183)

--------ACGGGAGGCTGCAGTCGAGAATCTTTCGCAATGGACGAAAGTCTGACGAAGCGACGCCGCGTGTGTGATGAAGGCCTTAGGGTTGTAAAGCACTTTCGCCTGGGAATAAGAGAGATTGGCTAATATCCAATCGATTTGAGCGTACCAGGTAAAGAAGCACCGGCTAACTCCGT---------

>Reference - Chlamydia abortus (Ov/B577)

-------TACGGAAGGCTGCAGTCGAGAATCTTTCGCAATGGACGAAAGTCTGACGAAGCGACGCCGCGTGTGTGATGAAGGCTCTAGGGTTGTAAAGCACTTTCGCTTGGGAATAAGAGAGATTGGCTAATATCCAATCGATTTGAGCGTACCAGGTAAAGAAGCACCGGCTAACTCCGT---------

>Reference - Chlamydia psittaci (6 BC)

-------TACGGAAGGCTGCAGTCGAGAATCTTTCGCAATGGACGAAAGTCTGACGAAGCGACGCCGCGTGTGTGATGAAGGCTCTAGGGTTGTAAAGCACTTTCGCTTGGGAATAAGAGAGATTGGCTAATATCCAATCGATTTGAGCGTACCAGGTAAAGAAGCACCGGCTAACTCCGT---------

>Reference - Parachlamydia acanthamoebae (Bn9)

-------TACGGGAGGCTGCAGTCGAGAATCATTCGCAATGGGCGAAAGCCTGACGATGCGACGCCGTGTGAGCGATGAAGGCCTTAGGGTCGTAAAGCTCTTTCGCCTGGGAGCAAGAGAAGCCGACTAATATTCGGCTAATTTGAGAGTACCAGGTAAAGAAGCACCGGCTAACTCCGT---------

>Reference - Neochlamydia hartmannellae (A1Hsp)

-------TACGGGAGGCTGCAGTCGAGAATCTTTCGCAATGGGCGAAAGCCTGACGAAGCGACGCCGTGTGAGCGATGAAGGCCTTCGGGTCGTAAAGCTCTTTCGCTTGGGAACAAGAGGATCTGTCTAATAAGCAGAGAATTTGAGGGTACCAGGTAAAGAAGCACCGGCTAACTCCGT---------

>Reference - Rhabdochlamydia crassificans (CRIB01)

-------TACGGGAGGCTGCAGTCGAGAATCTTTCGCAATGGGCGAAAGCCTGACGAAGCGACGCTGCGTGAGTGATGAAGGCTTTCGGGTTGTAAAACTCTTTCGCGCAGGAAAAAACCAAGCTTAGTGAATAACTAAATTTGGGACTAGTGATTGTACTGCGTAAAGAAGCACCGGCTAACTCCGT--

>Reference - Candidatus Protochlamydia naegleriophila (Knic)

-------TACGGGAGGCTGCAGTCGAGAATCATTCGCAATGGGCGAAAGCCTGACGATGCGACGCCGTGTGAGCGAAGAAGGCCTTAGGGTCGTAAAGCTCTTTCGCTTGGGAATAAGATAAACTGGCTAATATCCAGTAAGTTTGAACGTACCAGGTAAAGAAGCACCGGCTAACTCCGT---------

>Reference - Simkania negevensis (Z)

-------TACGGGAGGCTGCAGTCGAGAATCATTCGCAATGGGCGAAAGCCTGACGATGCGACGCCGTGTGAACGATGAAGGCCTTCGGGTTGTAAAGTTCTTTCGCTAAGGAACAAGAAAAGGTAACGAATAATTGCCTAATTTGAGGGTACTTGGTAAAGAAGCACCGGCTAACTCCGT---------

>Reference - Criblamydia sequanensis (CRIB-18)

-------TACGGGAGGCTGCAGTCGAGAATCTTTCGCAATGGGCGAAAGCCTGACGAAGCGACGCTGCGTGAGTGATGAAGGCCCTCGGGTCGTAAAGCTCTTTCGCCTGGGAACAAGGGAATCCGGCTAATATCCGGAGGACTTGAGAGTATCAGGTAAAGAAGCACCGGCTAACTCCGT---------

>Reference - Waddlia chondrophila (WSU 86-1044)

-------TACGGGAGGCTGCAGTCGAGAATCTTTCGCAATGGGCGAAAGCCTGACGAAGCGACGCCGTGTGAATGAAGAAGGCCCTTGGGTCGTAAAGTTCTTTCGCATGGGAACAAGAGAAGGATGCTAATATCATCTGGATTTGAGCGTACCTTGTAAAGAAGCACCGGCTAACTCCGT---------

>C1 - Uncultured Chlamydiales bacterium clone P1H10 ( 96.58 percent)

--------ACGGGAGGCTGCAGTCGAGAATCTTTCGCAATGGGCGCAAGCCTGACGAAGCGACGCCGTGTGGGTGATGACGGCCTTCGGGTTGTAAAGCCCTTTCGCTTGGGAACAAGAGACATTGGCTAATAACTAATTATTTGAGGGTACCA------------------------------------

>C10 - Uncultured Chlamydiales bacterium clone GE10193 (100 percent)

-------CACGGGAGGCTGCAGTCGAGAATCTTTCGCAATGGGCGCAAGCCTGACGAAGCGACGCCGTGTGGATGATGACAGCTTTCGGGTTGTAAAGTCCTTTCGCTTGGGAACAAGAGACATTAGCTAATAACTAATTATTTGAGGGTACCAGGTAAAGAAGCACCGGCTAACTCCAGA---------

>C12 - Uncultured Chlamydiales bacterium clone HE210045 C6 (90.16 percent)

----------------------GGGAGAATATTGCGCAATGGGCGAAAGCCTGACGAAAGCGACGCCGCGTGAGGGATGAAGGCCTTCGGGTTGTAAAGCTCTTTCGCGAGGGAACAAGAGAAGTGGATTAATAAGCCCCTGATTT--------------------------------------------

>C13 - Uncultured Chlamydiales bacterium clone 111 13 (97.63 percent)

---------CGGGAGGCTGCAGTCGAGAATCATTCGCAATGGGCGAAAGCCTGACGATGCGACGCCGTGTGAGCGATGAAGGCCTTCGGGTTGTAAAGCTCTTTCGCTTGGGAACAAGAGAAGTTGGCTAATATCCGGCTAATTTGAGGGTACCAGGTAAAGAAGCACCGGCTAACTCCTAGA-------

>C14 - Uncultured Chlamydiales bacterium clone 111 13 (98.14 percent)

----------------CTGCAGTCGAGAATCATTCGCAATGGGCGAAAGCCTGACGATGCGACGCCGTGTGAGCGATGAAGGCCTTCGGGTCGTAAAGCTCTTTCGCTTGGGAACAAGAAAAGTTGGCTAATATCCAGCTAATTTGAGGGTACCAGGTAAAGAAGCACCGGCTAACTACT----------

>C3 - Uncultured Chlamydiales bacterium clone VS30055biof (98.77 percent)

----------------CTGCAGTCGAGAATCATTCGCAATGGGCGCAAGCCTGACGATGCGACGCCGTGTGAGCGATGAAGGCCTTCGGGTTGTAAAGCTCTTTCGCTTGGGAACAAGAGAGGGTGACTAATAATCATCTAATTTGAGGGTACCAGGTAAAGAAGCACCGGCTAACTC------------

>C4 - Uncultured Chlamydiales bacterium clone VS30055biof (98.84 percent)

--------ACGGGAGGCTGCAGTCGAGAATCATTCGCAATGGGCGCAAGCCTGACGATGCGACGCCGTGTGAGCGATGAAGGCCTTCGGGTTGTAAAGCTCTTTCGCTTGGGAACAAGAGAGGGTGACTAATAATCATCTAATTTGAGGGTACCAGGTAAAGAAGCACCGGCTAACTCCAGAGC------

>C5 - Uncultured Chlamydiales bacterium isolate Otu001911 (80 percent)

---------ACGGGAGGCAGCAGTGGGGATTTTGGACAATGGGGGCAACCCTGATCCAGCCATGCCGCGTGAGTGAAGAAGGCCTTCGGGTTGTAAAGCTCTTTCAGACGGAAAGAAAACGGTTAGGTTAATACCCTGACTGGATGACGGTACTGTCAAAAGAAGCACCGGCTAACTCCAGAGT------

>C6 - Uncultured Chlamydiales bacterium isolate Otu001911 (80 percent)

------------GGAGGCAGCAGTGGGGATTTTGGACAATGGGGGCAACCCTGATCCAGCCATGCCGCGTGAGTGAAGAAGGCCTTCGGGTTGTAAAGCTCTTTCAGACGGAAAGAAAACGGTTAGGTTAATACCCTGACTGGATGACGGTACTGTCAAAAGAAGCACCGGCTAACTCCTGAGAG-----

>C8 - Uncultured Chlamydiales bacterium clone 111 13 (94.74 percent)

------------GGCTTTGGGTCGGAAAGCTCTTTCGCTTGGGAACAAGAGAGGTTGGCTAATATCCCGCTAATTTGAGGGTACCAGGTAAAGAAGCCCCGGCTAACTCCA-------------------------------------------------------------------------------

>C9 - Uncultured Chlamydiales bacterium clone GE10193 (98.82 percent)

---------ACGGGAGGCTGCAGTCGAGATCTTTCGCAATGGGCGCAAGCCTGACGAAGCGACGCCGTGTGGATGATGACAGCTTTCGGGTTGTAAAGTCCTTTCGCTTGGGAACAAGAGACATTAGCTAATAACTAATTATTTGAGGGTACCAGGTAAAGAAGCACCGGGCTAACTCAAGATT------

>I10 - Uncultured Chlamydiales bacterium clone GE10193 (82.64 percent)

-----------------------------TCTTCCAATGGGGCAAAGCCCGGCCAAACCAACGCCGGGGGGAGAAGAACGGCTTTCGGGTTGTAAGGTCCTTTCGCTGGGAAACAGGAAAGATTACTAAAAATCTAATTATTTGAGGGTACCAGGTAAAGAAGCACCGGCTAACTCCAA-----------

>I11 - Uncultured Candidatus Protochlamydia sp. clone OTU 133 (97.01 percent)

------------GAGGCTGCAGTCGAGAATCATTCGCAATGGGCGAAAGCCTGACGATGCGACGCCGTGTGAGCGAAGAAGGCCTTAGGGTCGTAAAGCTCTTTCGCCTCGGAACAAGAGAGGTTGGCTAATACCCAACTGATTTGAGCGTACGAGGTAAAGAAGCACCGGCTAACTCCCAAC-------

>I17 - Uncultured Chlamydiales bacterium clone GE11093water (89.88 percent)

------------------TGCAGTCGAGATCATTCGCACTGGGCGAAAAGCCTGGCAATGCGACGGCGTGTGGGCGAAGAAGGGCTTAGGGTCGTAAAGCTCTTTCGCTTGGGAACAAGAGAGGTTGGCTAATATCCCACTGAATGTTGAGACGTACCACGGTAAAGAAGCACCGGCTAACTCCACAA--

>I24 - Uncultured Chlamydiales bacterium clone SU16A10ocu (80.11 percent)

--CAACCTACGGGAGGCAGCAGTGGGGAATCTTGGACAATGGGGGCAACCCTGATCCAGCAATGCCGCGTGGGTGAAGAAGGCCTTCGGGTTGTAAAGCCCTTTCGGCAGGGAAGAAATCGGCGGGGCGAATAACCCTGCTGGATGACGGTACCTGCAAAAGAAGCACCGGCTAACTCCTAGACT-----

>I3 - Uncultured Chlamydiales bacterium clone rial (95.09 percent)

----------GGGGAGGCTGCAGTCGAGATCTTTCGCAATGGGCGAAAGCCTGACGAAGCGACGCTGTGTGAGCGATGAAGGCCTTAGGGTTGTAAAGCTCTTTCGCTTGGGAACAAGAGAAGTCAACTAATAATTGGCTAATTTGAGGGTACCAGGTAAAGAAGCACCGGCTAACTCCC----------

>L11 - Uncultured Chlamydiales bacterium isolate Otu001911 (88.37 percent)

------------------------------CTTGCGACAATGGGCGCAAGCCTGATCCAGCGACGCCGCGTGGGTGAAGAAGGCCTTCGGGTTGTAAAGCCCTTTCACCGGGGACGATGAT---------------------------------------------------------------------

>L12 - Uncultured Chlamydiae bacterium clone HTM866S-B26 (82.27 percent)

----------------GCAGCAGTGGGGATATTGCACAATGGGCGAAAGCCTGATGCAGCGACGCCGCGTGAGGGATGACGGCCTTCGGGTTGTAAACCTCTTTCAGCAGGGACGAAGCGCAAGTGACGGTACCTGCAAAAGAAGCACCGGCTAACTC--------------------------------

>L13 - Uncultured Chlamydiales bacterium clones 111 13 (96.79 percent)

----------------------TCGAGAATCATTCGCAATGGGCGAAAGCCTGACGATGCGACGCCGTGTGAGCGATGAAGGCCTTAGGGTCGTAAAGCTCTTTCGCTTGGGAACAAGAAAAGCTGGCTAATATCCAGCAGATTTGAGGGTACCAGGTAAAGAAGCACCGGCTAACTCC-----------

>L16 - Uncultured Chlamydiales bacterium clone 134 13 (100 percent)

-----------GGAGGCTGCAGTCGAGAATCATTCGCAATGGGCGAAAGCCTGACGATGCGACGCCGTGTGAGCGATGAAGGCCTTAGGGTCGTAAAGCTCTTTCGCTTGGGAACAAGAGAAGCTGACAAATAATCAGCTAATTTGAGGGTACCAGGTAAAGAAGCACCGGCTAACTC------------

>L22 - Uncultured Chlamydiae bacterium clone Paddy 16 5232 (94.7 percent)

---------------------------GATCATTCGCAATGGGCGAAAGCCTGACGAAGCGACGCCGTGTGAGCGATGAAGGCCTTAGGGTTGTAAAGCTCTTTCGCTTGGGAACAAGATACGCCGGCTAATATCTGGCGAATTTGAGAGTACCAGGTAAAGAAGCACCGGCTAACTCCAAA--------

>L23 - Uncultured Chlamydiae bacterium clone HTM866S-B26 (84.83 percent)

-----------GGAGGCAGCAGTGGGGAATATTGCACAATGGGCGGAAGCCTGATGCAGCGACGCCGCGTGAGGGATGAAGGCCTTCGGGTTGTAAACCTCTTTCAGCAGGGACGAAGCGTGAGTGACGGTACCTGCAAAAGAAGCACCGGCTAACTCCCAAAG--------------------------

>L25 - Uncultured Chlamydiales bacterium clone GE11061 (84 percent)

-----------------------------TCTTTCCCCATGGGCGACAGCCTGGCCAAACGACCCCCTGGGGGAGAAGAAGGCCTTCCGGGTGGAAAGCTCTTTCCCCTGGGAACAAGAAAAGGTAGCTAATAACTGATGAATTTGAGAGTACCAGGTAAAGAAGCACCGGCTAACTCC-----------

>L6 - Uncultured Parachlamydiaceae bacterium clone Ga4-sred-OTU-148 (87.80 percent)

------------------------------TATTGCACATGGGCGGAACCCTGATGCAGCAACGCCGCGTGCGGGATGACGGCCTTCGGGTTGTAAACCCCTTTCGCCTGGGACGAAGCGTGAGTGACGGATATGGGTGATGAAGCACCGGCTAACTCCAAGAAGCACCGGCTAACTCCA----------

>L7 - Uncultured Chlamydiales bacterium clone 12-91 (89.74 percent)

--------------------------------------ATGGGGGCGAGCCCTGTCCCGCATCGCCGCGGGCGGTATAAAGGCTTTCGGTTTGTAAACCCCTTTCGCCTGGGAGAAATCGGGAGGGACGATAATGCGGCTGGATGCACCGGCTTGGTCCAAGAACCCCCGTTTACTTCCA----------

>L8 - Uncultured Chlamydiales bacterium isolate Otu001911 (86.21 percent)

-----------------------------CTTGCGACAATGGGGGCAACCCTGATCCAGCTATGCCGCGTGGGTGAAGAAGGCCTTCGGGTTGTAAAGCCCTTTCGGCAGGGAAGAAATCGGCGGG----------------------------------------------------------------

>L9 - Uncultured Chlamydiales bacterium isolate Otu001911 (89.36 percent)

-----CAATCTCGGGAGGCAGCAGTGGGGAATCTTGGACAATGGGGGCAACCCTGATCCAGCCATGCCGCGTGTATGAAGAAGGCCTTCGGGTTGTAAAGTCCTTTCGGTAGGGAAGAAAACCTTTTG--------------------------------------------------------------
